# Transcriptome analysis of North American sweet birch (*Betula lenta*) revealed a higher expression of genes involved in the biosynthesis of secondary metabolites than European silver birch (*B. pendula*)

**DOI:** 10.1007/s10265-021-01343-y

**Published:** 2021-09-09

**Authors:** Kiran Singewar, Birgit Kersten, Christian R. Moschner, Eberhard Hartung, Matthias Fladung

**Affiliations:** 1 Institute of Agricultural Process Engineering, Christian-Albrechts University of Kiel, Max-Eyth- Str. 6, 24118 Kiel, Germany; 2 Thuenen-Institute of Forest Genetics, Sieker Landstraße 2, 22927 Grosshansdorf, Germany

**Keywords:** Secondary metabolites, Silver birch, Sweet birch, Transcription regulators, Transcriptomics

## Abstract

The North American *Betula lenta* L. (sweet birch) has been used for medicinal reasons for centuries by native Americans. Although sophisticated technologies have rapidly been developed, a large information gap has been observed regarding genetic regulators of medicinally important compounds in sweet birch. Very little is known on the different genes involved in secondary metabolic biosynthesis in sweet birch. To gain a deeper insight into genetic factors, we performed a transcriptome analysis of each three biological samples from different independent trees of sweet and European silver birch (*B. pendula* Roth). This allowed us to precisely quantify the transcripts of about 24,000 expressed genes including 29 prominent candidate genes putatively involved in the biosynthesis of secondary metabolites like terpenoids, and aromatic benzoic acids. A total number of 597 genes were differentially expressed between *B. lenta* and *B. pendula*, while 264 and 210 genes showed upregulation in the bark and leaf of *B. lenta*, respectively. Moreover, we identified 39 transcriptional regulatory elements, involved in secondary metabolite biosynthesis, upregulated in *B. lenta*. Our study demonstrated the potential of RNA sequencing to identify candidate genes interacting in secondary metabolite biosynthesis in sweet birch. The candidate genes identified in this study could be subjected to genetic engineering to functionally characterize them in sweet birch. This knowledge can be beneficial to the increase of therapeutically important compounds.

**Supplementary Information:**

The online version contains supplementary material available at 10.1007/s10265-021-01343-y.

## Introduction

The genus *Betula* (birch)—one of the dominating woody plant species of the Northern Hemisphere—incorporates diverse species with a wide range of morphological, genetic, and physiological variations. Despite their conventional economic benefits, many species are of medicinal and pharmacological importance (Ebeling et al. 2014; Rastogi et al. 2015; Yin et al. 2012, 2017). Above all, sweet birch (*Betula lenta* L.), also known as black and cherry birch has elite importance in ancient therapeutics (COSEWIC 2006; Sharik and Burton 1971; Stephens and Waggoner 1970). A forest study suggests sweet birch is ample in Massachusetts, Connecticut, New York, and Pennsylvania (Fernald et al. 1958; Lamson 1990). Sweet birch was introduced into the landscape in 1759 (Leak 1965; Lorimer 1980). The medicinal use of *B. lenta* by the native Americans is well documented (Gilmore 1933). Birch sap can be combined with corn and fermented to make beer (Suryawanshi 2000). Previously it was the only source of wintergreen oil extraction since it has the aroma of wintergreen emanatingfrom crushed bark and leaves (Ashburner and McAllister 2013; Singewar et al. 2020a, b). Extensive harvesting of sweet birch caused to become it endangered until the 1950–1970s (Leak 1965).

Sweet birch is a diploid and deciduous woody plant species with 28 numbers of chromosomes (2n) (Ashburner and McAllister 2013; Wang et al. 2016). It is closely related to *B. alleghaniensis*, the yellow birch (Sharik and Burton 1971). Crossing between *B. lenta* and *B. alleghaniensis*, and successful production of hybrids has been recorded in the past with low vigor and seed germination rates in F1 offspring (Sharik and Burton 1971). *B. jackii* is a natural hybrid of *B. lenta* and *B. pumila* which occurred at the Arnold Arboretum (Jack 1895).

Among the many other taxonomical distributions of the genus *Betula* (Furlow 1990; Winkler 1904), De Jong (1993) classified *B. lenta* into the subgenus *Betulenta*. Further, in the most recent resolved classification, *B. lenta* is subdivided in the section *Lentae* of the subgenus *Aspera.* (Ashburner and McAllister 2013; Wang et al. 2016). Various phylogenetic analyses demonstrated that the subgenus *Betulenta* to be among the oldest (Bina et al. 2016; De Jong 1993). Also, many evolutionary and population studies have been conducted in the genus *Betula* (Bina et al. 2016; Li et al. 2005; Singewar et al. 2020a; Wang et al. 2016). The previous (Bina et al. 2016) and most recent network analysis (Singewar et al. 2020a) suggest that the diploid *B. lenta* is one of the ancestors of the genus *Betula*. Similar network analysis also supports the study where *B. lenta* including *B. lenta* var. *uber* forms the oldest clade (Bina et al. 2016).

Many species of the genus *Betula* are polyploid, the chromosome count differs from 2n = 2x = 28 to 2n = 12x = 168 and ranges from diploid to dodecaphonic (Ashburner and McAllister 2013). The de novo genome of European silver birch (*Betula pendula* Roth) has been sequenced which is a diploid organism with 28 chromosomes (2n) having a haploid genome size of about 440 megabase pairs (Salojärvi et al. 2017). According to our knowledge, *B. pendula* and *B. platyphylla* (Chen et al. 2021) are the only reference genomes available publicly, and the only genome of *B. pendula* available on the genome browser (https://genomevolution.org/coge/GenomeInfo.pl?gid=35080) in the genus *Betula*. According to a PubMed database of NCBI (https://pubmed.ncbi.nlm.nih.gov/) survey, a large gap has been observed in the genetics and genomics of sweet birch (Singewar 2020; Zoladeski and Hayes 2013).

Knowingly or unknowingly, humans have been ignoring the medicinal importance of the sweet birch. The genetic factors involved in the secondary metabolite biosynthesis that makes *B. lenta* a pharmacologically important forest tree, remain unknown. Considering the species conservation and pharmacological importance of sweet birch, it represents a relevant target for genetic and genomic studies. Here, we profiled transcriptomes of *B. lenta* leaf and bark separately and performed a comparative analysis with *B. pendula*. Transcriptome analysis showed about 24,000 expressed genes including 29 prominent candidate genes putatively involved in the biosynthesis of secondary metabolites including terpenoids, aroma, and benzoic compounds. Moreover, 39 transcriptional regulatory elements involved in secondary metabolite biosynthesis were upregulated in *B. lenta*.

## Materials and methods

### Plant material and growth conditions

*Betula lenta* and *Betula pendula* were selected for RNA sequencing out of 29 species based on previous molecular genetic studies (Singewar 2020; Singewar et al. 2020a, b). RNA sequencing of leaf and bark tissues of both species was performed as a basis for comparative gene expression analysis to understand the tissue-specific gene expression. In April 2017, seeds were germinated in normal soil and a natural environment without any fertilizer in the poly-house at the Institute of Agricultural Process Engineering, Kiel University, Germany. Plantlet cultivation was carried out in a glasshouse at the Thuenen-Institute of Forest Genetics, Grosshansdorf, Germany, under identical conditions for all plantlets. In July 2019, leaf and bark tissues were harvested from the three different biological replicates of each species originated from three different mother trees, representing three different genotypes per species for RNA sequencing, which resulted in 12 samples.

### RNA extraction and sequencing

Total RNA was extracted from leaves and bark of *B. lenta* and *B. pendula*, described in Table 1 using the CTAB protocol of Dumolin et al. (1995). Three different leaves and three sections of bark per biological replicate were collected. To remove DNA contaminations, the Invitrogen Ambion Turbo DNA-free Kit (Fisher Scientific GmbH, Schwerte, Germany) was used following the manufacturer’s instructions. The quantity of RNA was determined with a Nanodrop 1000 spectrophotometer (Thermo Fisher Scientific, Wilmington, USA), A260/A280 readings > 2.0, and A260/A230 > 1.9). The RNA degradation and potential contamination were determined using agarose gel electrophoresis. Finally, the quality was measured with the Bioanalyzer Agilent 2100 (Agilent Technologies, Waldbronn, Germany). Samples matching the criteria (Table S1) were sent to Novogene Bioinformatics Technology Co., Ltd. (Hong Kong). For each species, three samples per biological replicate with the best quality values were accumulated. During the library preparation, the RNA is reverse transcribed to complementary DNA (cDNA). Sequencing was completed on the Illumina HiSeq 4000 platform to create 150 base pairs paired-end reads (on average 65 million read pairs per sample, Table S2).

**Table. 1:** Birch individuals used in this study

Species name	Source of material	Distribution	2n	Subgenus	Section
*Betula lenta*	BG Giessen, Germany	North America	2n	*Aspera*	*Lentae*
*Betula pendula*	Reinke Baumschulen, Germany	Europe and East Asia	2n	*Betula*	*Betula*

The table describes the source of the material and geographical distribution of the birch species. The ploidy condition and taxonomical position were decided according to Wang et al. (2016) and Ashburner and McAllister (2013)

*BG* Botanical garden

### Raw data filtering and bioinformatics workflow

The annotated reference genome of *B. pendula* (Salojarvi et al. 2017) was used as a reference for bioinformatic analysis of the RNA-seq data of all 12 samples. The Fastq files of the raw reads were trimmed and filtered using Trimmomatic v0.35 (Bolger et al. 2014) following parameters: ILLUMINACLIP: <fastaWithAdapters> :2:30:10 LEADING:3 TRAILING:3 SLIDINGWINDOW: 4:20 MINLEN: 50. Specific adapter sequences are given in Table S3. We mapped and quantified the filtered reads *versus* the *B. pendula* reference genome (version 1.4c) using the TopHat2 (Kim et al. 2013) with the mismatch = 2 parameter. Only filtered reads were used to analyze the mapping status of RNA-seq data to the reference genome. Further, the median number of reads mapped to the genome inside the window was calculated and transformed to the log2value. Gene expression level was measured by transcript abundance and estimated by counting the reads that mapped to the annotated genes or exons of the reference genome. The mapping results in BAM format were visualized in the integrative genomics viewer (IGV).

Gene expression level was measured by transcript abundance. The greater the abundance, the higher is the gene expression level. The Fragments Per Kilobase Million (FPKM) method was used to compare the gene expression levels of different genes which considers the effects of sequencing depth and gene length (Trapnell et al. 2010). HTSeq software was used to analyze the gene expression levels, using the union mode. The FPKM values > 1 are set as the threshold for determining whether the gene is expressed or not. Further, Bioconductor’s package DESeq (Wang et al. 2010) was used to normalize the read counts using the negative binomial distribution model and to determine differentially expressed gene screening (Robinson and Oshlack 2010). Differentially expressed genes were defined as having an adjusted p-value < 0.01 and an absolute log2 fold change > 1.5.

### GO and KEGG pathway network enrichment analyses

GO enrichment analysis was performed by GO seq (Young et al. 2010), which is based on Wallenius non-central hypergeometric distribution. All genes of the *B. pendula* reference genome with assigned GO terms were downloaded as a reference set. Differentially expressed genes (DEGs) were identified in the different comparisons and DEGs were used as test sets. GO terms with at least two differentially expressed genes in the test set were considered for the enrichment analyses. Further, Bioconductor’s topGO tool was used for testing GO terms while accounting for the topology. GO terms with a corrected p-value of *p* < 0.05 (hypergeometric statistical test with Bonferroni multiple comparison corrections) were selected as significantly enriched in the test set. To identify the putative biological function of genes, KEGG (Kyoto Encyclopedia of Genes and Genomes) curated database for functional orthologs (KO) was used.

### Transcription factor analysis

According to a previous study (Yang et al. 2012), various transcription factors (TF) from different families participate in the secondary metabolite regulation. Here, we screened the putative TFs in the differentially expressed genes and considered only those genes for further analysis which were enriched in GO and KEGG pathways. Furthermore, an additional survey was carried out to consider all possible TFs. In that, amino acid sequences of previously functionally studied TFs were used as BLAST queries (Table S4) to perform BLASTp search (1E−5) versus all annotated proteins in the *B. pendula* genome (https://genomevolution.org/). Eventually, the resulting hits were screened in the differentially expressed as well as GO and KEGG enriched genes. Significantly upregulated genes in *B. lenta* were selected for comparative analysis.

## Results

### Differentially expressed genes

The overall distribution of differentially expressed genes was inferred through the volcano plots with the following thresholds, padj < 0.01 and absolute log2 fold change > 1.5. A total number of 597 genes were differentially expressed between *B lenta* and *B. pendula*. Around 123 and 474 genes showed up- and down-regulation, respectively. A total number of 877 genes were differentially expressed between the bark tissue of *B. lenta* and *B. pendula*, respectively. Around 264 and 613 genes showed up- and down-regulation, respectively. Similarly, 210 and 807 genes were up- and down-regulated, respectively in *B. lenta* leaves when compared to *B. pendula* ones, respectively.

### The co-expression of genes in the different comparisons

Gene expression level is measured by transcript abundance. The greater the abundance, the higher is the gene expression level. In our RNA-seq analysis, the gene expression level is estimated by counting the reads that map to genes or exons (Table S5). The read count is not only proportional to the actual gene expression level but is also proportional to the gene length and the sequencing depth. For the gene expression levels estimated from different genes and experiments to be comparable, the FPKM is used (Table S6).

**Fig. 1:**
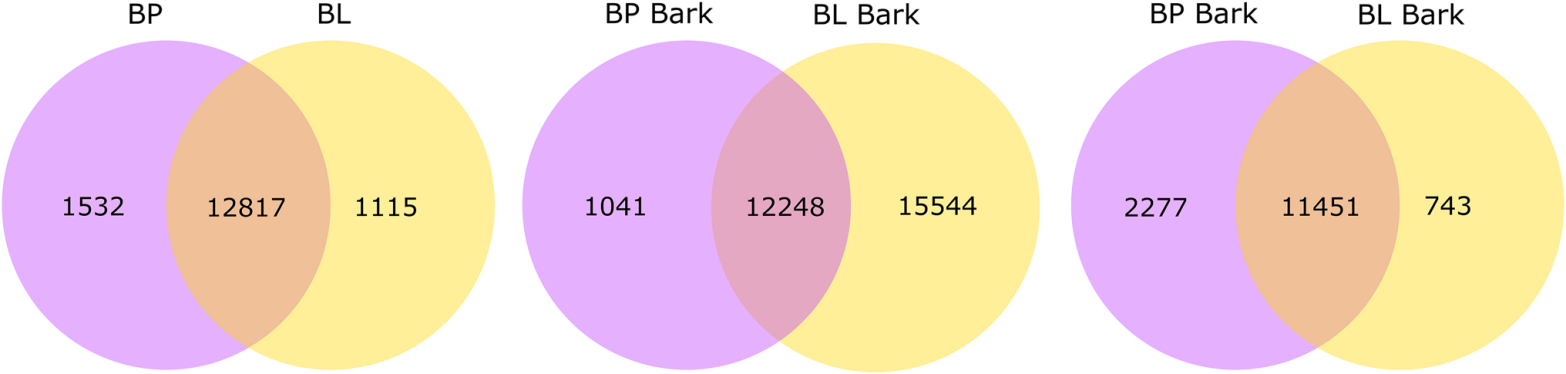
Graphical representation of a total number of co-expressed genes. The sum of the numbers in each circle is the total number of genes expressed within a sample, and the overlap represents the genes expressed in common between samples

A total number of 1532 or 1115 genes were expressed in *B. pendula* only or *B. lenta* only, respectively (expressed genes with FPKM values > 1). In total, 12,817 genes were co-expressed in the analyzed tissues of both species under the experimental conditions. The number of genes expressed in the bark and leaf of *B. pendula* only or *B. lenta* only were 1041 or 1544, and 2277 or 743, respectively (Fig. 1).

### GO enrichment analysis of differentially expressed genes

**Fig. 2:**
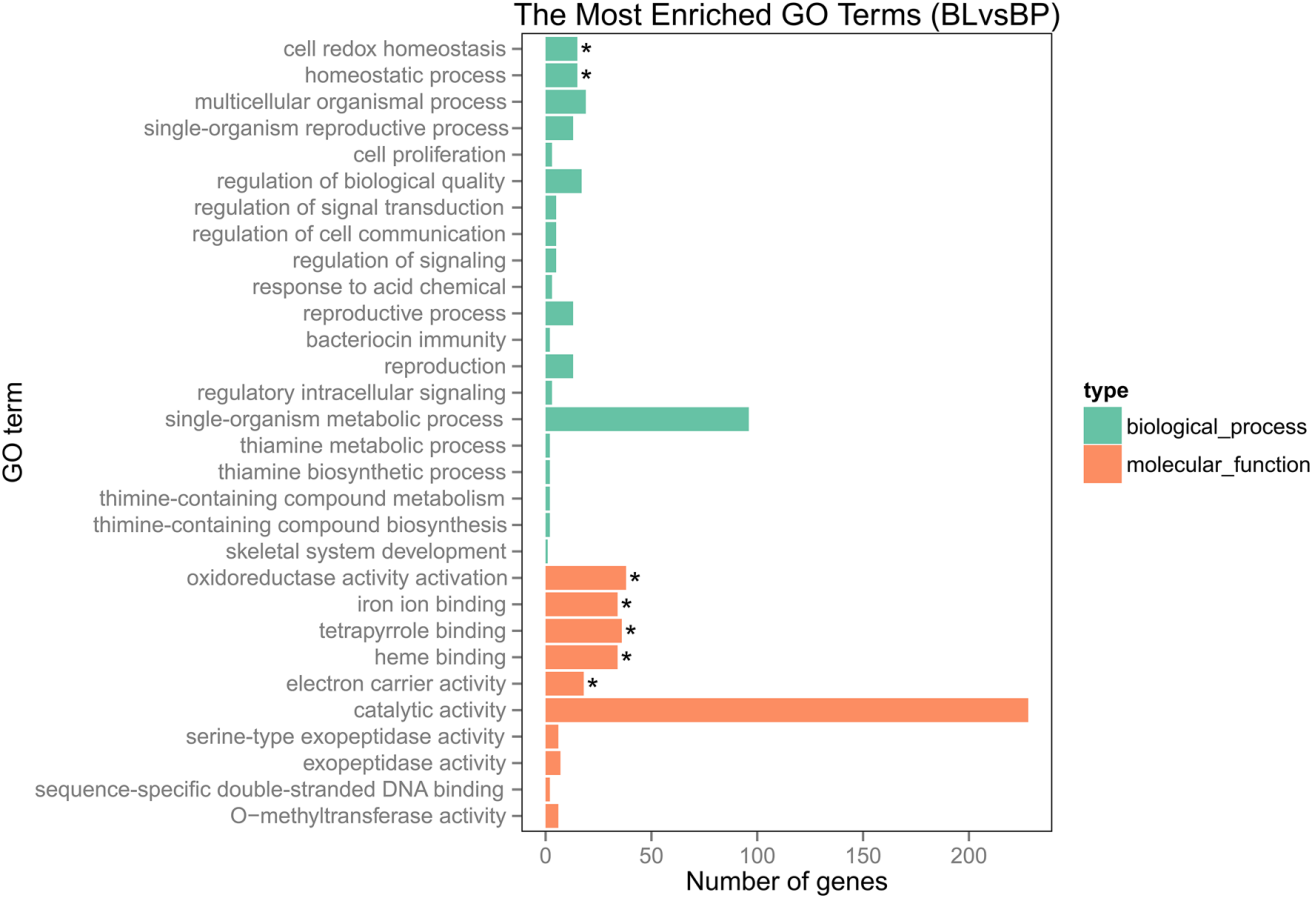
GO annotation of differentially expressed genes between *B. lenta* and *B. pendula* and GO enrichment analysis. The most frequent 30 GO terms with at least 2 assigned differentially expressed genes are shown. The x-axis is the number of differentially expressed genes assigned to the respective GO term that is shown at the y-axis. In the GO enrichment analysis, all genes of the *B. pendula* reference genome were used as a reference set. Most of the differentially expressed genes were categorized in ‘biological process’ and ‘molecular function’. Different colors are used for biological process and molecular function, in which the enriched GO terms are marked by “*”

A GO enrichment bar chart is used to illustrate functional GO annotation of differentially expressed genes in *B. lenta* versus *B. pendula* (Fig. 2). The most frequent 30 GO terms comprising all GO terms assigned to more than two differentially expressed genes are shown and significantly enriched GO terms are marked (with an asterisk). The GO term ‘catalytic activity’ showed the highest frequency in the set of differentially expressed genes. Significant enrichment of genes potentially involved in the biological processes of “cell redox homeostasis” and “homeostatic process” with predicted molecular functions of “oxidoreductase activity” and “iron ion binding” among others was obvious in the set of differentially expressed genes in *B. lenta* versus *B. pendula*.

**Fig. 3:**
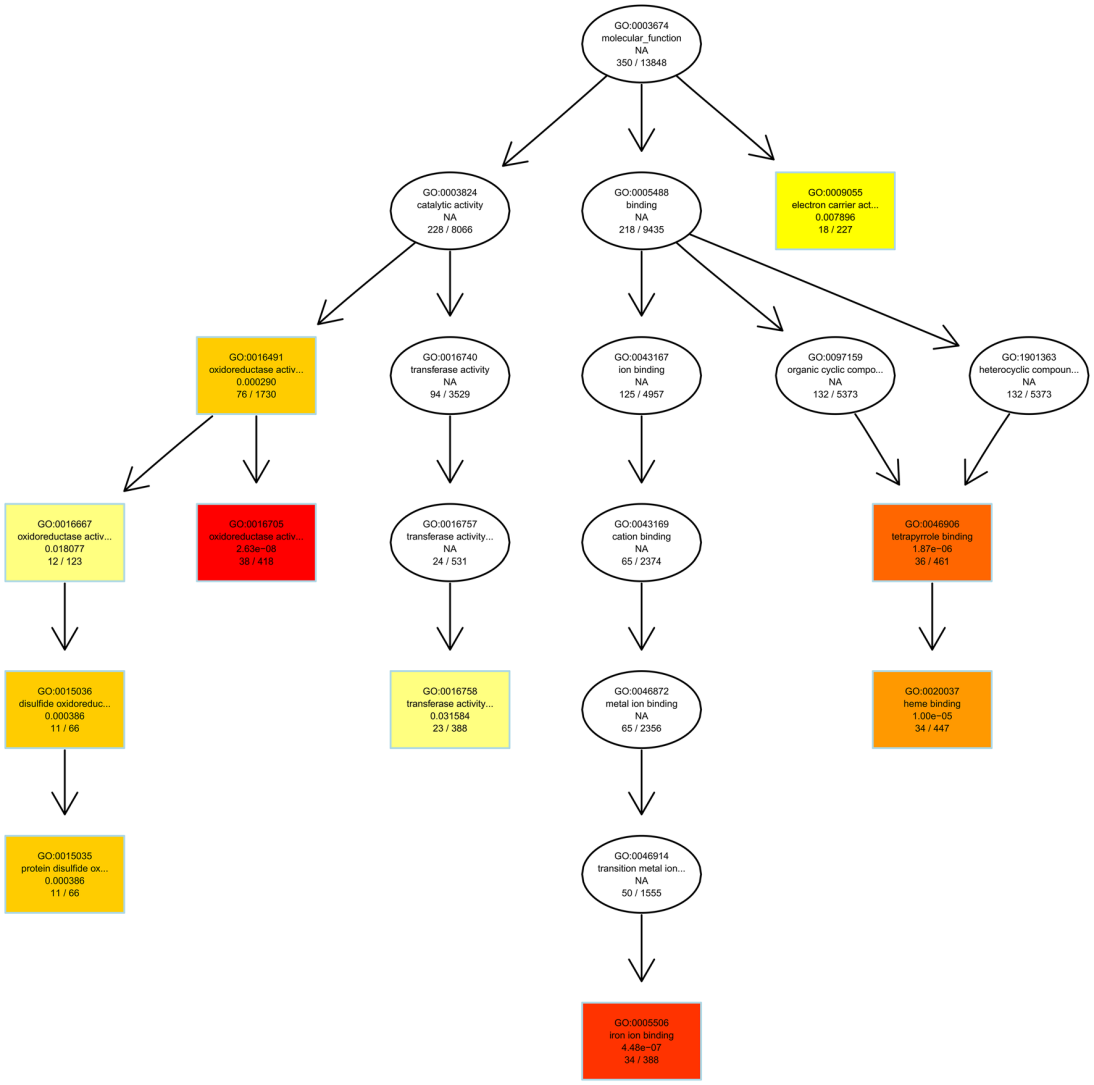
The GO terms of the GO main class “molecular function” enriched in the set of differentially expressed genes in *B. lenta* versus *B. pendula.* Each node represents a GO term and significantly enriched GO terms are boxed. The darker the color is, the lower is the p-value and the higher is the enrichment level of the GO term in the enrichment test. The name and corrected p-value of each term are present on the node

Further, the directed acyclic graph (DAG) was used to show the results of GO enrichment of DEGs (Fig. 3). The branches represent the containment relationships, and the range of functions gets smaller and smaller from top to bottom. Generally, the top ten of GO enrichment results is selected as the master nodes in a DAG, showing the associated GO terms together via the containment relationship, and the degree of colors represent the extent of enrichment. In this study, DAG figures of biological process and molecular function are drawn. Here, the DAG figure of molecular function represents differentially expressed genes in *B. lenta* (Fig. 3). DAG figures of biological process are provided in supplementary data (Fig. S1).

### KEGG enrichment analysis

**Fig. 4:**
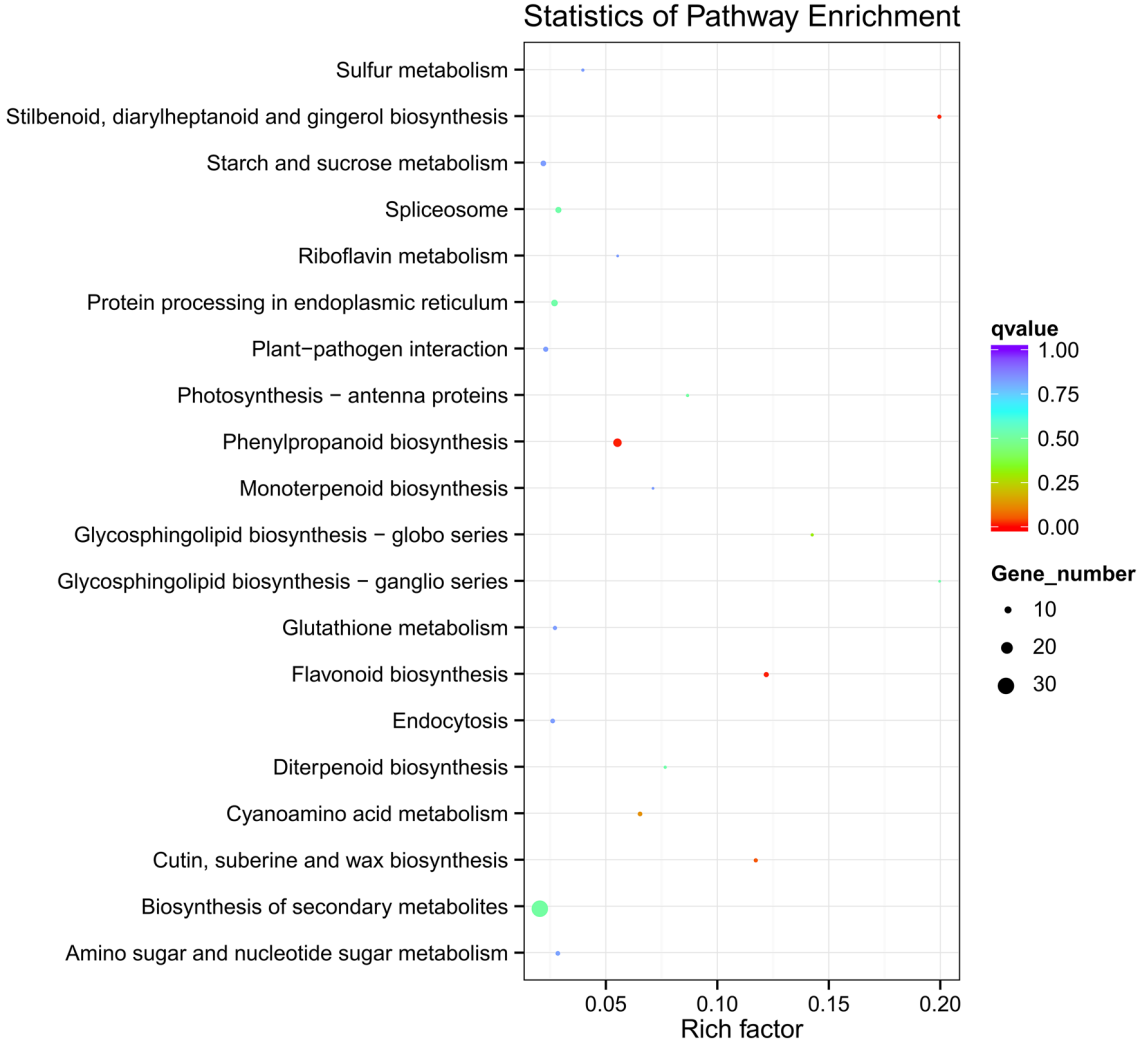
The y-axis shows the name of the pathway, and the x-axis shows the rich factor. Dot size represents the number of different genes, and the color indicates the q-value The color of the circle represents the q-value; a smaller q-value indicates higher reliability for the significance of differential expression of genes in this pathway

In the scatter plot, the enrichment degree of KEGG was measured through the rich factor, Q value, and gene counts enriched by this pathway. The top 20 most significantly enriched pathways are chosen in KEGG scatter plot (Fig. 4). Genes involved in the biosynthesis of secondary metabolites were shown to be most expressed in KEGG analysis. Additionally, genes involved in plant-pathogen interactions, flavonoid biosynthesis, phenylpropanoid biosynthesis, and cyanoamino acid metabolism were shown to be enriched (Fig. 4).

### Identification of genes involved in secondary metabolites and aromatic compounds

**Fig. 5:**
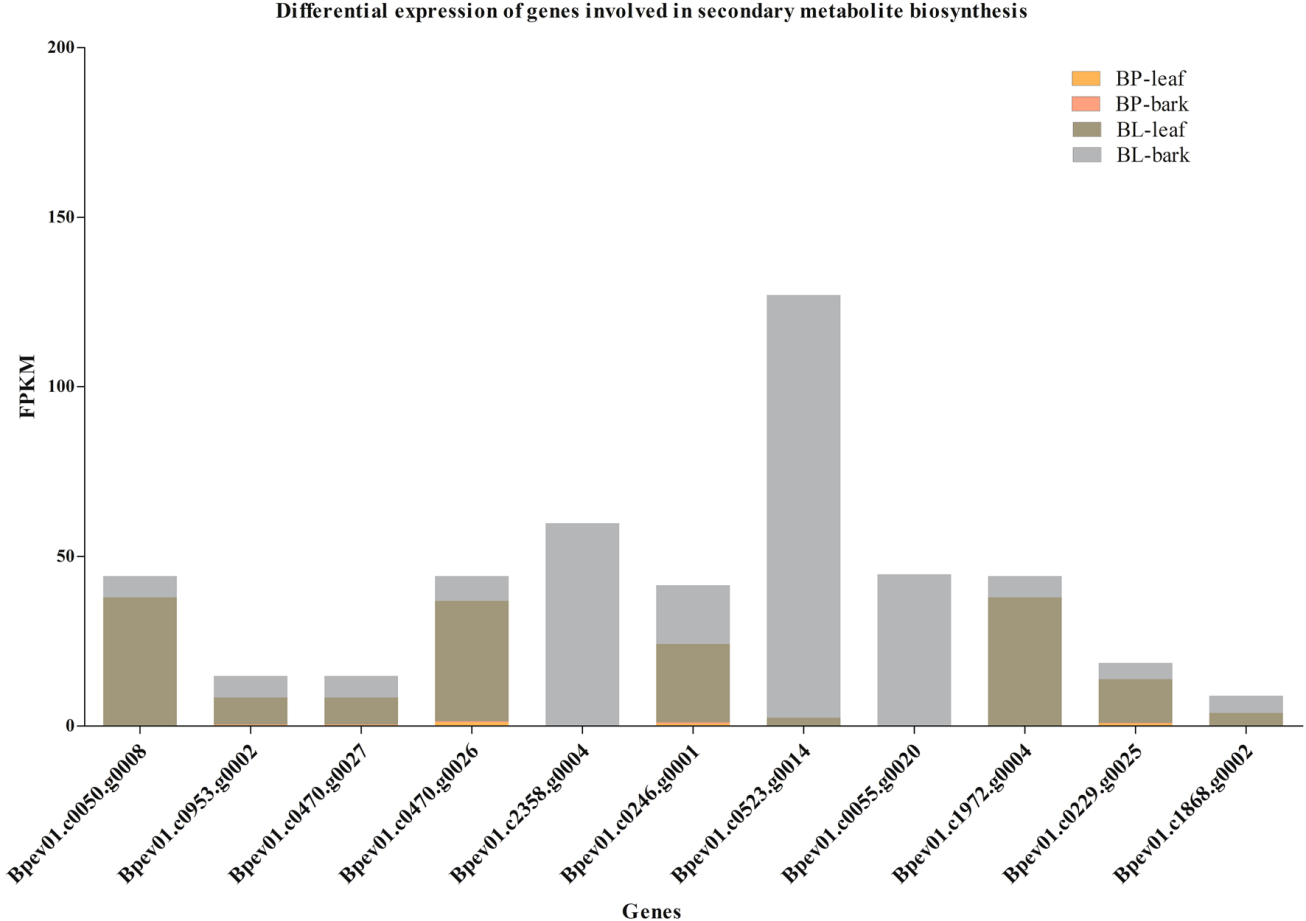
Putative genes involved in secondary metabolite biosynthesis. A total number of 11 genes possibly involved in secondary metabolite biosynthesis were identified. All the genes showed upregulated expression in the bark and leaf tissue of *B. lenta* when compared t*o B. pendula*

**Fig. 6:**
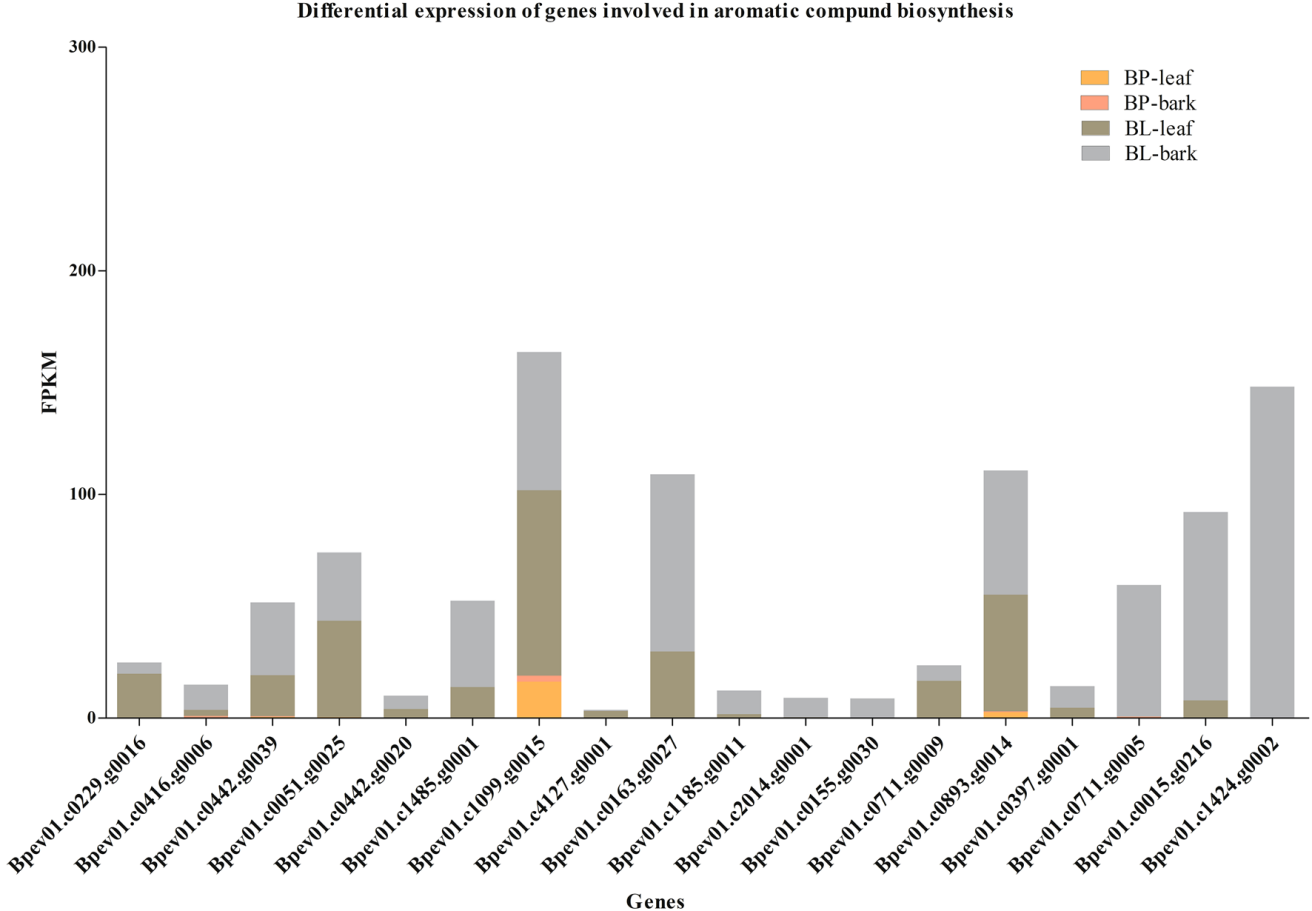
Putative genes involved in aromatic compound biosynthesis. A total number of 18 genes involved in aromatic compound biosynthesis were identified. All the genes showed upregulation in bark and leaf tissues of *B. lenta* while low or no expression in *B. pendula*

Most of the differentially expressed genes were from the GO term, catalytic activity, and from the KEGG pathway enrichment analysis, biosynthesis of secondary metabolites in the total transcriptome data. All the differentially expressed genes in *B. lenta* that were assigned to enriched GO terms and/or KEGG pathways were further analyzed. A total number of 11 and 18 genes involved in secondary metabolism and aromatic compounds were upregulated in *B. lenta*, respectively (Figs. 5 and 6). Almost all the genes showed higher expression in the bark tissues compared to the leaf. Gene identifiers and their designated annotations were collected (Table S7).

### Identification of transcription factors (TF) involved in secondary metabolism

**Fig. 7:**
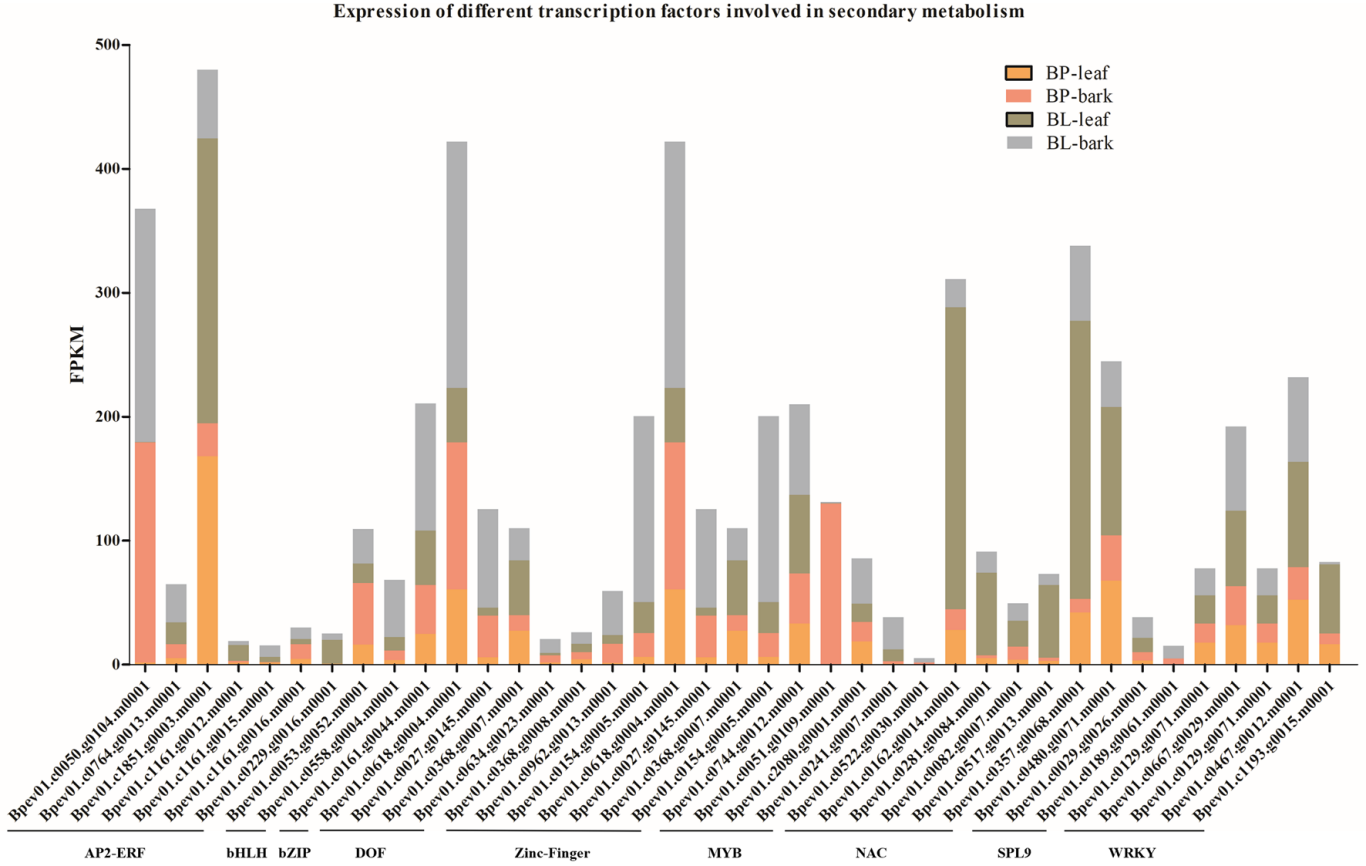
Different transcription factors involved in secondary metabolism: A total number of 39 transcription factors identified showed upregulation in *B. lenta* versus *B. pendula*. All the identified genes are present on the x-axis, while transcript abundance is displayed on the y-axis

In plants, transcription factors (TFs) play a vital role in gene regulation that could also cause metabolic variability through interaction with the promoter region of a gene. The amino acid sequences of previously described transcription factors (Table S4) were used to identify the TF in *B. pendula* and *B. lenta*. All transcription factors identified among the *B. pendula* gene models which are in the list of upregulated DEGs in *B. lenta* were considered for further analysis. 39 putative *B. lenta* transcription factors belonging to nine TF families were identified (Fig. 7). Nine families of TF that are supposed to be involved in secondary metabolic biosynthesis including AP2-ERF, bHLH, bZIP, DOF, Zinc-finger, MYB, NAC, SPL9 and, WRKY, were detected. Every TF showed upregulated expression in leaf and bark tissue of *B. lenta* compared to *B. pendula*. Among them, *AP2-ERF* and *Zinc-finger* were the most abundant TF family (7 genes each), followed by *NAC* (6 genes), *WRKY* (5 genes), *MYB* and *DOF* (4 genes each), *SPL9* (3 genes), *bHLH* (2 genes) and *bZIP* (1 gene).

The *APETALA2/ethylene response factor* (*AP2/ERF*) family are the TFs that could be characterized by their DNA-binding AP2 domain. The domain consists of around 60 conserved amino acid residues (Mizoi et al. 2012). Further, zinc-finger TFs functions in gene regulation in stably transformed plants. The target of the *zinc-finger* TF is the *Arabidopsis thaliana APETALA3* (*AP3*) gene, involved in floral organ identification (Xuen et al. 2002). The *NAC* TF families were reported to be a regulator of camalexin, which is a plant secondary metabolite. A study has shown that *WRKY* TF is also taking part in the secondary metabolite volatile terpene biosynthesis (Skibbe et al. 2008). The expression of DNA-binding-with-one-finger (DOF) TF is studied to be in response to pathogens and the phytohormone jasmonic acid as a part of regulatory networks (Skirycz et al. 2006). The vital function of *MYB* TF is to regulate secondary metabolites including terpenoids (Galis et al. 2006; Yin et al. 2017). The *SPL9* (*SQUAMOSA Promoter Binding Protein-Like*) participates in a broad range of developmental processes like anthocyanin accumulation which is important for pigments (Jin-Ying et al. 2011). Pathogen defense and stress signaling is regulated by TF *bZIP* (Jakoby et al. 2002). Functional genomics investigation of these TFs can allow us to understand the regulatory networks involved in the secondary metabolite biosynthesis in *B. lenta*.

## Discussion

*Betula lenta* is extensively known as a traditional medicinal plant (Ebeling et al. 2014; Rastogi et al. 2015; Yin et al. 2012, 2017). According to authors knowledge and online research, *B. lenta* has largely been ignored by the modern generation in the past century. According to initial olfactory and monographic evidence (Ashburner and McAllister 2013; Singewar et al. 2020a, b), it contains abundant secondary metabolites including aromatic compounds and response regulators. Fortunately, the reference genomes of *B. pendula* and *B. platyphylla* are available (Chen et al. 2021; Salojärvi et al. 2017). Furthermore, *B. pendula* is the only species in the genus *Betula* whose genome is available on the web genome browser (https://genomevolution.org/), providing an opportunity to explore other therapeutically important and largely ignored species of the genus. Although the cost of sequencing technology has reduced drastically which is a good sign for more scientific research, a recent survey on available genome sequences of the species within the genus *Betula* in the NCBI database clearly shows the scarcity of knowledge within the subgenus *Aspera* (Singewar 2020; Zoladeski and Hayes 2013). We performed the transcriptome analysis of *B. lenta* in comparison to *B. pendula* to reduce the information gap.

Here, we performed high-throughput transcriptome sequencing for bark and leaf tissues of *B. lenta*. We utilized three different biological replicates of each species, originated from three different seeds each, representing three different genotypes per species. The comparative analysis of these divergent *Betula* genotypes resulted in the novel insights of the least studied woody plant species, *B. lenta* in comparison to *B. pendula*. A total number of 24,554 genes were identified to be expressed in the experimental dataset considering 28,153 genes annotated in the reference genome. When considering the set of upregulated DEGs in the *B. lenta* and *B. pendula* comparative analysis, several significantly enriched GO terms assigned to the GO main classes like biological processes and molecular functions were identified (Fig. 2). Within the molecular function category, the vast majority of genes were related to catalytic activity, following the abundant secondary metabolites produced in *B. lenta*. Further, 18 genes were upregulated in *B. lenta* as assigned to the enriched GO term ‘aromatic compounds biosynthesis process’ in the main class biological processes (Fig. 6).

Through mapping the genes onto the KEGG pathways, 11 genes were discovered to be assigned to the enriched pathway ‘biosynthesis of secondary metabolites’ (Fig. 4). Among them, two genes have a putative function in shikimate dehydrogenase, four in beta-glucosidase, and five each involved in pyruvate dehydrogenase (PDH), cytochrome methyltransferase, gibberellin 2 oxidase, bioactive compounds, and disease resistance, respectively in *B. lenta* (Figs. 5 and 6). Genes from shikimate dehydrogenase have drawn great attention from researchers due to their special aromatic amino acid biosynthesis (Tzin and Galili 2018).

The identified 11 and 18 genes involved in secondary metabolism and biosynthesis of aromatic compounds were respectively, further subjected to NCBI BLAST search for their putative function confirmation (Table S7). The identification and annotation have provided the origin for analyzing specific pathways in *B. lenta*. These genes were involved in shikimate dehydrogenase, terpenoid backbone biosynthesis, and aromatic compounds including those encoding important proteins and regulatory elements (Gilchrist and Kosuge 1980; Tzin and Galili 2018). Unfortunately, the experimental validation of these novel genes was not part of the current study. Therefore, further functional characterization of these new and innovative findings will improve our understanding of the molecular mechanisms underlying aromatic amino acid and terpenoid biosynthesis that are important for therapeutics. Functional characterization of these genes will be vital to confirm the activity as well as use in therapeutics.

**Fig. 8:**
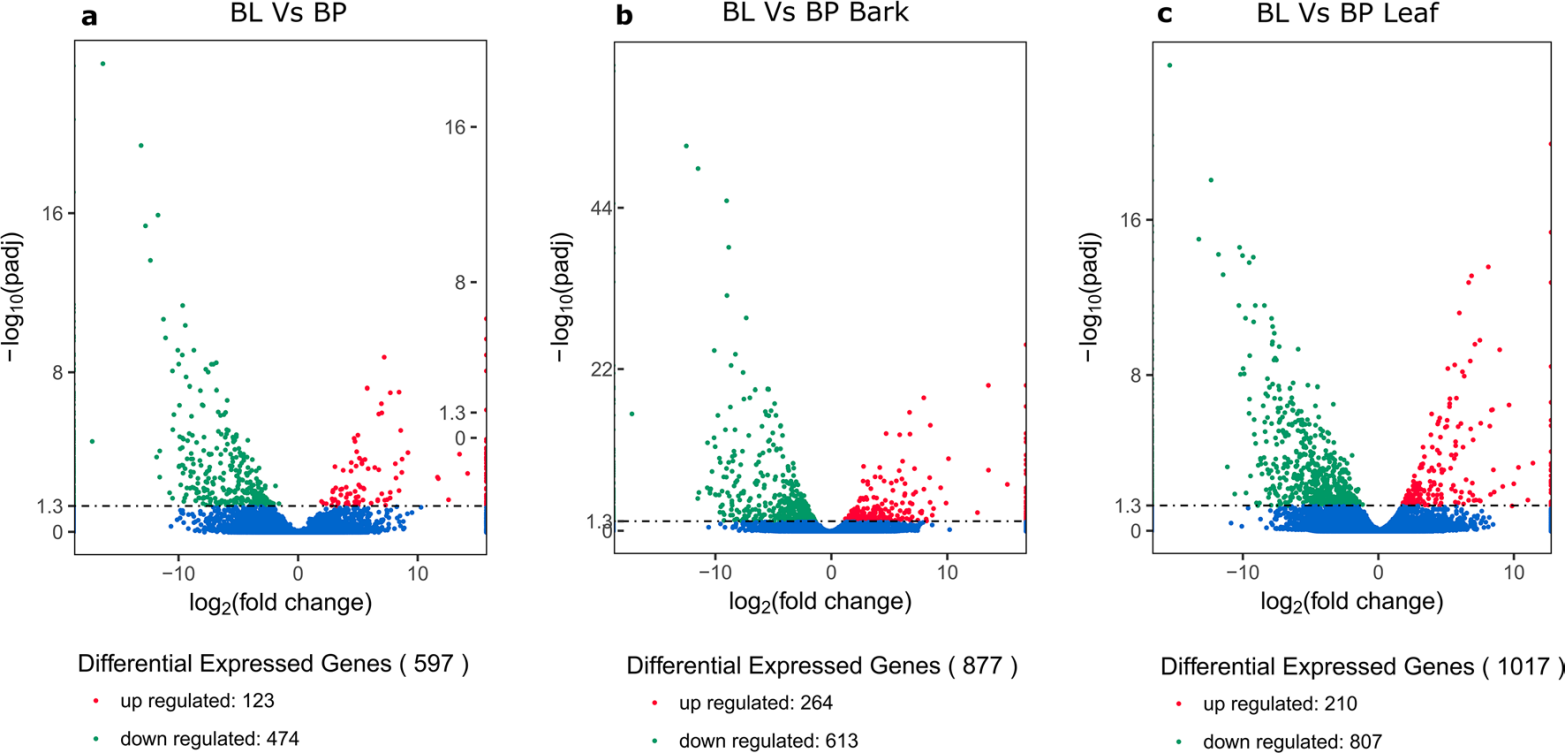
Volcano plot showing differentially expressed genes in three different experimental comparisons. The x-axis shows the fold_2_ (fold change) in gene expression between different samples, and the y-axis shows the statistical significance of the differences. Three different comparative analyses were carried out showed in **a–c** (BL: *B. lenta*; BP: *B. pendula*). Significantly up- and down-regulated genes in both species (adjusted *p* < 0.01 and absolute log2 fold change > 1.5) are highlighted in red and green, respectively. The dashed line indicates the p-value significance threshold. Blue dots represent genes that did not express differently in the comparison shown

In addition, our study examined the differentially expressed genes between the bark and leaf based on FPKM values (Fig. 8). The results suggested that several genes were uniquely expressed in either bark or leaf tissues and many genes were expressed at different levels. Further study on these DEGs combined with metabolomes will enable us to more clearly understand the biosynthetic process of secondary metabolites.

TFs affect the metabolic flux by regulating gene expression. In this work, a total of 39 TFs specifically upregulated in *B. lenta* were identified, including bHLH (2), AP2/ERF (7), MYB (4), DOF (4), NAC (6), SPL9 (3), and WRKY (5) families (Fig. 7). Many studies have shown that these TFs play noteworthy roles in modulating the biosynthesis of secondary metabolites. For example, the bHLH transcription factor, AabHLH1 in *Artemisia annua*, constructively controls the biosynthesis of artemisinin (Ji et al. 2014). The AP2/ERF members of ORCA2 and ORCA3 in *Catharanthus roseus* hold together with the promoter of strictosidine synthases (STR) to tune the terpenoid indole alkaloid metabolism (van der Fits et al. 2001). It is significant to subject all these TF for the gene-editing methods to detect the TFs for regulating different aromatic amino acid and terpenoid biosynthesis in *B. lenta*. Further research on these TFs will be beneficial for the alteration of these metabolic pathways and eventually for escalating the production of secondary metabolites with medicinal value in *B. lenta*.

## Conclusions and outlook

In this study, we presented the importance of transcriptome sequencing of the therapeutically important forest tree species *B. lenta*. A total number of 24,000 expressed genes were listed and several differentially expressed genes were annotated by the GO and KEGG database, referring to different plant metabolic pathways and biosynthesis processes. The identified candidate genes by KEGG and GO database are vital to understanding important regulators of secondary metabolite biosynthesis. Here, we also focused on searching for candidate genes involved in aromatic compound biosynthesis, in that 29 genes were involved in this bioprocess. The transcriptome information presented in our study also revealed that various genes are involved in the biosynthetic pathways of phenylpropanoid, flavonoids. Additionally, our study identified several transcription factors related to the biosynthesis of secondary metabolites in *B. lenta*. Taken together, the transcriptome data generated in our study allowed for discovering novel genes involved in specific secondary metabolic pathways and provide the basis for improving the yields of valuable metabolites in plants by metabolic engineering. Moreover, it is also highly valuable to pave the way for functional and comparative genomic studies of this promising medicinal plant *B. lenta* in the future. Unfortunately, biological validation of the unique genes was not feasible in the frame of this study. Experimental affirmation of the study is the next step and planned in the second part of the study.

## Supplementary Information

Below is the link to the electronic supplementary material.

Supplementary material 1 (PDF 456 kb)

## Data Availability

The RNA-seq data set has been deposited in NCBI as BioProject under the number PRJNA756395.
