# Supplementary material for: Transcriptome analysis of North American sweet birch (Betula lenta) revealed a higher expression of genes involved in the biosynthesis of secondary metabolites than European silver birch (B. pendula)
Source: J Plant Res. 2021 Sep 9;134(6):1253–64. doi: 10.1007/s10265-021-01343-y (PMC8514364; doi:10.1007/s10265-021-01343-y)
Supplement: Supplementary file 1 — Supplementary material 1 (PDF 456 kb) [file 10265_2021_1343_MOESM1_ESM.pdf]

Electronic supplementary materials

Title: Transcriptome analysis of North American sweet birch (*Betula lenta*) revealed a higher expression of genes involved in the biosynthesis of secondary metabolites than European silver birch (*B. pendula*)

Authors:

Kiran Singewar[1,2], Birgit Kersten[2], Christian R. Moschner[1], Eberhard Hartung[1], Matthias Fladung[2*]

[1]Institute of Agricultural Process Engineering, Christian-Albrechts University of Kiel, Max-Eyth-Str. 6, 24118 Kiel, Germany.

[2]Thuenen-Institute of Forest Genetics, Sieker Landstraße 2, 22927 Grosshansdorf, Germany.

Journal:

Journal of Plant Research

Corresponding author:

Thuenen-Institute of Forest Genetics, Sieker Landstraße 2, 22927 Grosshansdorf, Germany.

Tel: +49 4102 696 0

Fax: +49 4102 696 200

E-mail: matthias.fladung@thuenen.de

Content:

Tables S1–S7

Fig. S1

Table S1: Total number of RNA probes, Qubit, RIN values, volume and concentration provided to Novogene

RNA Samples	RIN values	Volume	Concentration
BL_L-1	> 7.3	> 20µl	> 50ng/µl
BL_L-2	> 7.3	> 20µl	> 50ng/µl
BL_L-3	> 7.3	> 20µl	> 50ng/µl
BL_B-1	> 7.3	> 20µl	> 50ng/µl
BL_B-2	> 7.3	> 20µl	> 50ng/µl
BL_B-3	> 7.3	> 20µl	> 50ng/µl
BP_L-1	> 7.3	> 20µl	> 50ng/µl
BP_L-2	> 7.3	> 20µl	> 50ng/µl
BP_L-3	> 7.3	> 20µl	> 50ng/µl
BP_B-1	> 7.3	> 20µl	> 50ng/µl
BP_B-2	> 7.3	> 20µl	> 50ng/µl
BP_B-3	> 7.3	> 20µl	> 50ng/µl

Table S2: Obtained quality of generated sequence reads.

Sample name	Raw reads	Clean reads	Clean bases	Error rate (%)	Q20 (%)	Q30 (%)	GC content (%)
BL_B_1	68325850	66552840	10G	0.03	97.37	92.70	51.32
BL_B_3	76840266	75653430	11.3G	0.03	97.00	91.89	49.52
BL_B_2	63699184	61976190	9.3G	0.03	96.25	90.49	46.59
BL_L_1	62352090	60501508	9.1G	0.03	97.30	92.64	56.98
BL_L_3	75310566	74478920	11.2G	0.03	97.24	92.33	45.62
BL_L_2	62097834	60851924	9.1G	0.03	95.85	89.61	45.56
BP_B_3	58597510	57595638	8.6G	0.03	96.86	91.68	56.72
BP_B_1	65537926	63806712	9.6G	0.03	95.62	89.19	46.79
BP_B_2	66171056	64382578	9.7G	0.03	95.77	89.47	47.39
BP_L_1	61786034	60281084	9G	0.03	96.10	90.09	45.59
BP_L_2	62672438	61004342	9.2G	0.03	95.73	89.35	45.44
BP_L_3	72071074	69402714	10.4G	0.03	95.96	89.79	45.60

Table S3: Adapter information

Name	Part#	Sequence from 5' to 3'
RNA 5' Adapter	15013205	AATGATACGGCGACCACCGAGATCTACACTCTTTCCCTACACGACGCTCTTCCGATCT
RNA 3' Adapter	15013207	GATCGGAAGAGCACACGTCTGAACTCCAGTCAC (6-nucleotide index) ATCTCGTATGCCGTCTTCTGCTTG

Table S4: Amino acid sequences of previously functionally studied Transcription factors (TFs) were used as references are collected in a table.

Transcription factor	References
MYB	Teng et al 2005
MYC2	Hong et al 2012
AP2/ERF	Menke et al 1999
WRKY	Zheng et al 2006
Zinc finger	Pauw et al 2004
DOF	Skirycz et al 2007
NAC	Saga et al 2012

Table S5: Gene expression level was measured by transcript abundance.

Sample name	BL_B_1	BL_B_3	BL_B_2	BL_L_1	BL_L_3	BL_L_2
Total reads	66552840	75653430	61976190	60501508	74478920	60851924
Total mapped	15616427 (23.46%)	22986099 (30.38%)	22523130 (36.34%)	4404443 (7.28%)	31010573 (41.64%)	22320573 (36.68%)
Multiple mapped	1411802 (2.12%)	1454879 (1.92%)	576918 (0.93%)	633029 (1.05%)	692463 (0.93%)	811388 (1.33%)
Uniquely mapped	14204625 (21.34%)	21531220 (28.46%)	21946212 (35.41%)	3771414 (6.23%)	30318110 (40.71%)	21509185 (35.35%)
Reads map to '+'	7129506 (10.71%)	10722022 (14.17%)	10963718 (17.69%)	1888309 (3.12%)	15211026 (20.42%)	10795961 (17.74%)
Reads map to '-'	7075119 (10.63%)	10809198 (14.29%)	10982494 (17.72%)	1883105 (3.11%)	15107084 (20.28%)	10713224 (17.61%)
Non-splice reads	7904928 (11.88%)	11257430 (14.88%)	11039890 (17.81%)	2270148 (3.75%)	15254760 (20.48%)	10876028 (17.87%)
Splice reads	6299697 (9.47%)	10273790 (13.58%)	10906322 (17.6%)	1501266 (2.48%)	15063350 (20.22%)	10633157 (17.47%)

Sample name	BP_B_1	BP_B_3	BP_B_2	BP_L_1	BP_L_3	BP_L_2
Total reads	57595638	63806712	64382578	60281084	61004342	69402714
Total mapped	6955952 (12.08%)	43749820 (68.57%)	44323778 (68.84%)	43588880 (72.31%)	43543964 (71.38%)	48942254 (70.52%)
Multiple mapped	574713 (1%)	923104 (1.45%)	1032305 (1.6%)	589135 (0.98%)	545058 (0.89%)	818903 (1.18%)
Uniquely mapped	6381239 (11.08%)	42826716 (67.12%)	43291473 (67.24%)	42999745 (71.33%)	42998906 (70.48%)	48123351 (69.34%)
Reads map to '+'	3162393 (5.49%)	21481746 (33.67%)	21651632 (33.63%)	21526191 (35.71%)	21544180 (35.32%)	24103852 (34.73%)
Reads map to '-'	3218846 (5.59%)	21344970 (33.45%)	21639841 (33.61%)	21473554 (35.62%)	21454726 (35.17%)	24019499 (34.61%)
Non-splice reads	3942285 (6.84%)	27050880 (42.4%)	27439601 (42.62%)	24370180 (40.43%)	24245585 (39.74%)	27270316 (39.29%)
Splice reads	2438954 (4.23%)	15775836 (24.72%)	15851872 (24.62%)	18629565 (30.9%)	18753321 (30.74%)	20853035 (30.05%)

Table S6: The number of genes with different expression levels

FPKM Interval	BL_B_1	BL_B_3	BL_B_2	BL_L_1	BL_L_3	BL_L_2
0~1	11049(45.61%)	10992(45.38%)	10548(43.54%)	15363(63.42%)	12279(50.69%)	11546(47.66%)
1~3	1460(6.03%)	1713(7.07%)	2067(8.53%)	231(0.95%)	2022(8.35%)	1945(8.03%)
3~15	4599(18.99%)	4764(19.67%)	5247(21.66%)	2836(11.71%)	4061(16.76%)	4384(18.10%)
15~60	4772(19.70%)	4656(19.22%)	4396(18.15%)	3695(15.25%)	3831(15.81%)	4212(17.39%)
>60	2344(9.68%)	2099(8.66%)	1966(8.12%)	2099(8.66%)	2031(8.38%)	2137(8.82%)

FPKM Interval	BP_B_3	BP_B_1	BP_B_2	BP_L_1	BP_L_2	BP_L_3
0~1	14791(61.06%)	10641(43.93%)	10719(44.25%)	10810(44.63%)	10810(44.63%)	10716(44.24%)
1~3	247(1.02%)	1954(8.07%)	2185(9.02%)	1910(7.88%)	2029(8.38%)	1937(8.00%)
3~15	3303(13.64%)	5356(22.11%)	5729(23.65%)	4603(19.00%)	4526(18.68%)	4628(19.11%)
15~60	4059(16.76%)	4536(18.73%)	3982(16.44%)	4796(19.80%)	4725(19.51%)	4741(19.57%)
>60	1824(7.53%)	1737(7.17%)	1609(6.64%)	2105(8.69%)	2134(8.81%)	2202(9.09%)

Table S7: Identified genes putatively involved in secondary metabolite biosynthesis including terpenoids and aromatic compounds.

Gene identifiers	Designated annotations (NCBI)	Function	Ref
Bpev01.c0229.g0016	Ethylene_response_factor	a key regulatory hub, integrating ethylene, abscisic acid, jasmonate, and redox signaling in the plant response to several abiotic stresses	doi:10.1104/pp.15.00677
Bpev01.c0416.g0006	Serine/threonine kinase protein	are the cyclin-dependent kinases (CDKs), identified originally for their role in the control of the cell cycle	The yin and yang of protein phosphorylation and signaling
Bpev01.c0442.g0039	Copper-transporting_ATPase_1	transmembrane proteins	DOI: 10.1002/iub.1437
Bpev01.c0051.g0025	Ethylene_response_factor	a key regulatory hub, integrating ethylene, abscisic acid, jasmonate, and redox signaling in the plant response to several abiotic stresses	doi:10.1104/pp.15.00677
Bpev01.c0442.g0020	G-box_binding_factor_3	transcriptional activator in light-regulated expression	PubMed:22718771
Bpev01.c1485.g0001	SHORT_vEGETATIvE_PHASE	MADS-box gene SHORT VEGETATIVE PHASE (SVP) is a key regulator of two developmental phases. It functions as a repressor of the floral transition during the vegetative phase and later it contributes to the specification of floral meristems	https://doi.org/10.1186/gb-2013-14-6-r56
Bpev01.c1099.g0015	oligopeptide_transporter_4	Involved in the translocation of tetra- and pentapeptides across the cellular membrane in an energy-dependent manner.	https://doi.org/10.1104/pp.010332

Bpev01.c4127.g0001	receptor-like_protein_kinase_1	receptor with a dual specificity kinase activity acting on both serine/threonine- and tyrosine-containing substrates	https://doi.org/10.1073/pnas.0810249106
Bpev01.c0163.g0027	Heat_stress_transcription factor_C-1	Structure, Regulation, and Function in Response to Abiotic Stresses	https://doi.org/10.3389/fpls.2016.00114
Bpev01.c1185.g0011	Ethylene_response_factor	a key regulatory hub, integrating ethylene, abscisic acid, jasmonate, and redox signaling in the plant response to several abiotic stresses	doi:10.1104/pp.15.00677
Bpev01.c0155.g0030	DOF_zinc_finger_protein_1	The aromatic residues of the Dof domain may also play an important role	https://doi.org/10.1111/j.1365-313X.2003.01997.x
Bpev01.c0711.g0009	O-methyltransferase family_protein	Plant O-methyltransferases (OMTs) constitute a large family of enzymes that methylate the oxygen atom of a variety of secondary metabolites including phenylpropanoids, flavonoids, and alkaloids.	DOI: 10.1139/g07-077
Bpev01.c0893.g0014	UDP-Glycosyltransferase superfamily_protein	Has high affinity for 4-aminobenzoate (an aromatic amino-acid anion).	DOI:https://doi.org/10.1074/jbc.M709591200
Bpev01.c0397.g0001	transcription_activators IIF	positive regulation of transcription by RNA polymerase II,	DOI: 10.7554/eLife.00473
Bpev01.c0711.g0005	methyltransferase activity	Plant O-methyltransferases (OMTs) constitute a large family of enzymes that methylate the oxygen atom of a variety of secondary metabolites including phenylpropanoids, flavonoids, and alkaloids.	DOI: 10.1139/g07-077
Bpev01.c0015.g0216	methyl easterase	Involved in SA signaling. Demethylation of aromatic compounds/ floral scent metabolites like MeSA and MeJA.	DOI: 10.1093/pcp/pcx198<br>DOI: 10.1104/pp.108.118224
Bpev01.c1424.g0002	GDSL esterases secondary metabolites	The GDSL esterases/lipases are found throughout all kingdoms of life. Due to their broad substrate specificity, these highly promising enzymes can be potentially used for biotechnological application in a wide range of industries (e.g. food, fragrance, cosmetics, textile, pharmaceutical, and detergent industry)	https://doi.org/10.1016/j.plipres.2004.09.002
Bpev01.c0050.g0008	beta hexosaminidase	Hydrolyses monosaccharide residues	doi: 10.1155/2014/186029
Bpev01.c0953.g0002	Shikimate_dehydrogenase	The aromatic nature of shikimate pathway intermediates gives rise to a wealth of potential bio-replacements for commonly fossil fuel-derived aromatics, as	https://doi.org/10.3389/fbioe.2018.00032

		well as naturally produced secondary metabolites.	
Bpev01.c0470.g0027	Shikimate_dehydrogenase	The aromatic nature of shikimate pathway intermediates gives rise to a wealth of potential bio-replacements for commonly fossil fuel-derived aromatics, as well as naturally produced secondary metabolites.	https://doi.org/10.3389/fbioe.2018.00032
Bpev01.c0470.g0026	beta_glucosidase	catalyze the last and final step in cellulose hydrolysis. Cellulase enzymes hydrolyze the cellulose to produce cellobiose and other short oligosaccharides which are finally hydrolyzed to glucose by β-glucosidase.	https://doi.org/10.1007/s13205-015-0328-z
Bpev01.c2358.g0004	beta_glucosidase	catalyze the last and final step in cellulose hydrolysis. Cellulase enzymes hydrolyze the cellulose to produce cellobiose and other short oligosaccharides which are finally hydrolyzed to glucose by β-glucosidase.	https://doi.org/10.1007/s13205-015-0328-z
Bpev01.c0246.g0001	Pyruvate dehydrogenase (PDH)	involved in the decarboxylation of pyruvate and reductive acetylation of lipoic acid to form acetyl-CoA by catalysing the overall conversion of pyruvate to acetyl-CoA and CO 2	DOI:10.1046/j.1432-1033.2003.03469.x
Bpev01.c0523.g0014	beta_glucosidase	catalyze the last and final step in cellulose hydrolysis. Cellulase enzymes hydrolyze the cellulose to produce cellobiose and other short oligosaccharides which are finally hydrolyzed to glucose by β-glucosidase.	https://doi.org/10.1007/s13205-015-0328-z
Bpev01.c0055.g0020	Cytochrome/ Beta-amyrin 11-oxidase	Cytochrome P450s play critical roles in oxidative reactions during biosynthesis of diverse natural plant products. Beta-amyrin 11-oxidase (CYP88D6) is a cytochrome P450 which catalyzes two sequential oxidation steps of beta-amyrin at the position of C-11 to yield 11-oxo-beta-amyrin.	https://doi.org/10.1073/pnas.0803876105 And https://doi.org/10.1007/s12033-018-0082-7
Bpev01.c1972.g0004	methyltransferase activity	Plant O-methyltransferases (OMTs) constitute a large family of enzymes that methylate the oxygen atom of a variety of secondary metabolites including phenylpropanoids, flavonoids, and alkaloids.	DOI: 10.1139/g07-077

Bpev01.c0229.g0025	GIBBERELLIN_2-OXIDASE Bioactive compunds	Gibberellin 2-oxidase (GA2ox) plays a direct role in determining the levels of bioactive GAs by catalyzing bioactive GAs or their immediate precursors to inactive forms.	https://doi.org/10.3389/fpls.2017.02103
Bpev01.c1868.g0002	Disease_resistance protein RGA1	guard the plant against pathogens that contain an appropriate avirulence protein via a direct or indirect interaction with this avirulence protein.	https://www.uniprot.org/uniprot/Q7XA42

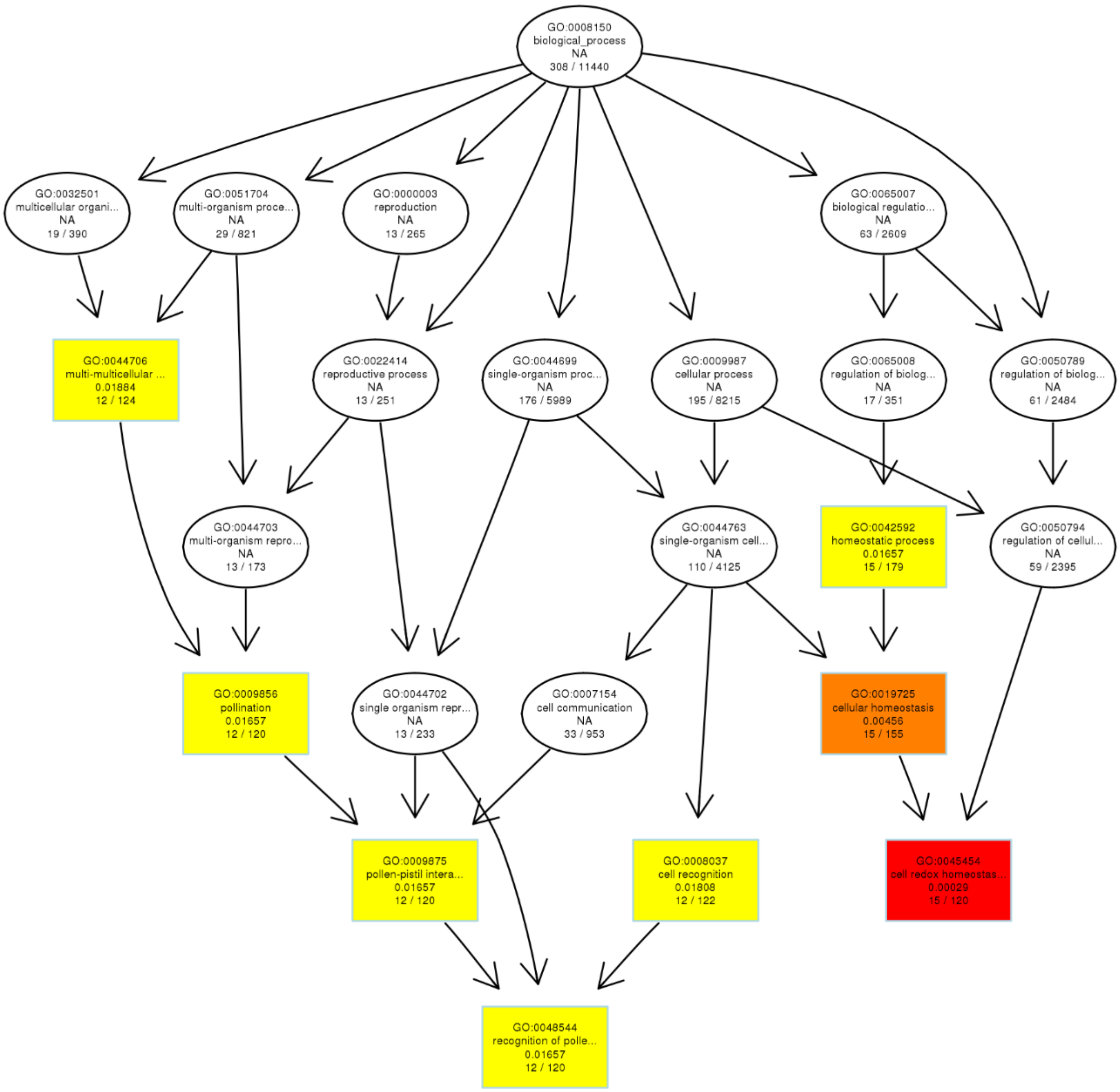

Figure S1: The GO terms of the GO main class "biological process" enriched in the set of differentially expressed genes in *B. lenta* versus *B. pendula*. Each node represents a GO term, and significantly enriched GO terms are boxed. The darker the color is, the higher is the enrichment level of the GO term in the enrichment test. The name and corrected p-value of each term are present on the node.
